# Supplementary material for: Effects of mass casualty incidents on anxiety, depression and PTSD among doctors and nurses: a systematic review protocol
Source: BMJ Open. 2023 Sep 11;13(9):e075478. doi: 10.1136/bmjopen-2023-075478 (PMC10496702; doi:10.1136/bmjopen-2023-075478)
Supplement: Supplementary data [file bmjopen-2023-075478supp001.pdf]

Supplementary Table

Table S1: PRISMA-P Statement – Checklist of items

Section and topic	Item No	Page number of manuscript
**ADMINISTRATIVE INFORMATION**		
Title:		
Identification	1a	1
Update	1b	N/A
Registration	2	2
Authors:		
Contact	3a	1
Contributions	3b	12
Amendments	4	N/A
Support:		
Sources	5a	6-7
Sponsor	5b	12
Role of sponsor or funder	5c	12
**INTRODUCTION**		
Rationale	6	3-6
Objectives	7	6
**METHODS**		
Eligibility criteria	8	6-7
Information sources	9	7
Search strategy	10	7
Study records:		
Data management	11a	8
Selection process	11b	8-9
Data collection process	11c	8-9
Data items	12	9-10
Outcomes and prioritization	13	6-7
Risk of bias in individual studies	14	9
Data synthesis	15a-15d	9-10
Meta-bias(es)	16	10
Confidence in cumulative evidence	17	10

Table S2: Comprehensive search strategy in PubMed

Sl no.	Search queries
#1	"Mass Casualty Incidents"[Mesh]) OR (MCI[Title/Abstract])) OR (explosion*[Title/Abstract])) OR (plane crash*[Title/Abstract])) OR (air crash*[Title/Abstract])) OR (air accident*[Title/Abstract])) OR (train derailment[Title/Abstract])) OR (train bombing[Title/Abstract])) OR (road traffic[Title/Abstract])) OR (bus bombing[Title/Abstract])) OR (bus crash*[Title/Abstract])) OR (car crash*[Title/Abstract])) OR (suicide bombing[Title/Abstract])) OR (bombing[Title/Abstract])) OR (terror attack[Title/Abstract])) OR (terrorism[Title/Abstract])) OR (bioterrorism[Title/Abstract])) OR (accident*[Title/Abstract])) OR (industrial accident*[Title/Abstract])) OR (fire*[Title/Abstract])) OR (factory fire*[Title/Abstract])) OR (chemical spill*[Title/Abstract])) OR (CBRNE accident*[Title/Abstract])) OR (building collapse[Title/Abstract])) OR (mass injur*[Title/Abstract])) OR (gunshot*[Title/Abstract])) OR (mass shoot*[Title/Abstract])) OR (massive chemical contamination[Title/Abstract])) OR (radiological dispersal device[Title/Abstract])) OR (dirty bomb*[Title/Abstract])) OR (emergenc*[Title/Abstract])) OR (volcanic eruption*[Title/Abstract]) OR ("Earthquakes"[Mesh])
# 2	"Stress Disorders, Post-Traumatic"[Mesh]) OR (PTSD[Title/Abstract])) OR ("Anxiety"[Mesh])) OR ("Anxiety Disorders"[Mesh])) OR ("Patient Health Questionnaire"[Mesh])) OR ("Depression"[Mesh])) OR ("Depressive Disorder"[Mesh])) OR ("Mental Health"[Mesh])) OR (mental health disorder*[Title/Abstract])) OR (psychological disorder*[Title/Abstract])) OR (psychological condition*[Title/Abstract])) OR (psychological distres*[Title/Abstract])) OR (psychological impact*[Title/Abstract])) OR (mental health outcome*[Title/Abstract])) OR (mental condition*[Title/Abstract])) OR (emotional impact*[Title/Abstract])
#3	"Nurses"[Mesh]) OR ("Nurses, Community Health"[Mesh])) OR ("Nurses, Public Health"[Mesh])) OR (emergency nurse*[Title/Abstract])) OR (prehospital nurse*[Title/Abstract])) OR (registered nurse*[Title/Abstract])) OR (intern nurse*[Title/Abstract])) OR (nurse in emergency department[Title/Abstract])) OR ("Physicians"[Mesh])) OR (medical doctor*[Title/Abstract])) OR (doctor*[Title/Abstract])) OR (emergency doctor*[Title/Abstract])) OR (prehospital doctor*[Title/Abstract])) OR (medical officer*[Title/Abstract])) OR (doctor in emergency department[Title/Abstract])
# 4	#1 AND #2 AND #3 AND
# 5	#1 AND #2 AND #3 AND Filter: English, Full text, Humans, from 2010 – 2022
