# Supplementary material for: Effects of mass casualty incidents on anxiety, depression and PTSD among doctors and nurses: a systematic review protocol
Source: BMJ Open. 2023 Sep 11;13(9):e075478. doi: 10.1136/bmjopen-2023-075478 (PMC10496702; doi:10.1136/bmjopen-2023-075478)
Supplement: Author's manuscript [file bmjopen-2023-075478.draft_revisions.pdf]

BMJ Open is committed to open peer review. As part of this commitment we make the peer review history of every article we publish publicly available.

When an article is published we post the peer reviewers' comments and the authors' responses online. We also post the versions of the paper that were used during peer review. These are the versions that the peer review comments apply to.

The versions of the paper that follow are the versions that were submitted during the peer review process. They are not the versions of record or the final published versions. They should not be cited or distributed as the published version of this manuscript.

BMJ Open is an open access journal and the full, final, typeset and author-corrected version of record of the manuscript is available on our site with no access controls, subscription charges or pay-per-view fees (http://bmjopen.bmj.com).

If you have any questions on BMJ Open's open peer review process please email info.bmjopen@bmj.com

BMJ Open

Effects of Mass Casualty Incidents (MCIs) on Anxiety, Depression, and PTSD among Nurses and Doctors: A Systematic Review Protocol

Journal:	*BMJ Open*
Manuscript ID	bmjopen-2023-075478
Article Type:	Protocol
Date Submitted by the Author:	09-May-2023

Complete List of Authors:	Uddin, Helal; Karolinska Institute, Global Public Health ; East West University,<br>Hasan, Md. Khalid; Dhaka University<br>castro delgado, rafael; University of Oviedo, Medicine. Public Health; SAMU-Asturias, Emergency Medicine

Keywords:	Anxiety disorders < PSYCHIATRY, Depression & mood disorders < PSYCHIATRY, Physicians, MENTAL HEALTH

SCHOLARONE™
Manuscripts

Effects of Mass Casualty Incidents (MCIs) on Anxiety, Depression, and PTSD among Nurses and Doctors: A Systematic Review Protocol

Helal Uddin[1,2,3], Md Khalid Hasan[4], and Rafael Castro Delgado [2,5]

[1]Department of Global Public Health, Karolinska Institutet, Solna, 17177, Sweden

[2]Department of Medicine, University of Oviedo, 33006 Oviedo, Spain

[3]Department of Sociology, East West University, Dhaka 1212, Bangladesh

[4]Institute of Disaster Management and Vulnerability Studies, University of Dhaka, Dhaka 1000, Bangladesh

[5]Health Service of the Principality of Asturias (SAMU-Asturias), Health Research Institute of the Principality of Asturias (Research Group on Prehospital Care and Disasters, GIAPREDE), 33001, Oviedo, Spain

Author Note:

Helal Uddin https://orcid.org/0000-0002-0767-3174

Md. Khalid Hasan https://orcid.org/0000-0002-9293-9693

Rafael Castro Delgado https://orcid.org/0000-0001-9520-656X

Corresponding Author:

Helal Uddin, Department of Global Public Health, Karolinska Institutet, Solna, 17177, Sweden. Email: md.helal.uddin@stud.ki.se; helal@ewubd.edu

&

Rafael Castro Delgado, Health Service of the Principality of Asturias (SAMU-Asturias), Health Research Institute of the Principality of Asturias (Research Group on Prehospital Care and Disasters, GIAPREDE), 33001, Oviedo, Spain. Email: castrorafael@uniovi.es; rafacastrosamu@yahoo.es

Word count: Text=2383, Abstract =234

Number of tables & figures: Table 2, Figure 1, Supplementary Table 2

For peer review only - http://bmjopen.bmj.com/site/about/guidelines.xhtml

Abstract

Introduction: Both nurses and doctors showed a greater risk of being exposed to different mental health conditions following mass casualties. This systematic review aims to synthesize the existing evidence on the prevalence of anxiety, depression, and PTSD and their associated risk factors among nurses and doctors following mass casualty incidents.

Methods and analysis: Seven electronic databases (PubMed, PsycINFO, MEDLINE Ovid, Embase, CINAHL, Web of Science, and Nursing & Allied Health database) will be searched from 2010 to 2022 with peer-reviewed full-text in English language using the pre-defined keywords. Two reviewers will screen the titles, abstracts, and full texts using the eligibility criteria and extract data independently. The STROBE Checklist for observational studies, COREQ Checklist for qualitative studies, and the Mixed Methods Appraisal tool (MMAT) for mixed method studies will be used to measure the quality appraisal of eligible studies. A third reviewer will resolve the discrepancies when the two reviewers cannot reach an agreement in any step. The result from the eligible studies will be described following narrative synthesis with the key characteristics and findings of the included studies, and meta-analysis will be performed if applicable.

Ethics and dissemination: This systematic review deals with existing published studies without any personally identifiable information of participants. Therefore, ethical approval from the research committee is not required. Findings from this review will be disseminated in peer-reviewed journals and presented at relevant international conferences.

PROSPERO registration number: CRD42023412852

Keywords: mass casualty incident, anxiety, depression, PTSD, nurse, doctor

Strengths and limitations

For peer review only - http://bmjopen.bmj.com/site/about/guidelines.xhtml

- To the best knowledge, this is the first systematic review on two crucial medical responders (emergency nurses and doctors), and their three mental health outcomes (anxiety, depression, and PTSD) following MCIs.
- Another biggest strength of this review is to strictly follow the PRISMA guidelines and use standard quality assessment tools.
- Above and beyond, the review will incorporate studies irrespective of location or geography.
- However, excluding gray literature and including studies published only in English will miss some potential studies that may be a source of publication bias.
- As different authors use heterogeneous tools to measure the outcomes, there will be a possibility of reporting bias.

1. Introduction

Mass casualty incidents (MCIs), and disasters are frequently used interchangeably in the literature, although they describe specific conditions and entities. From a medical perspective, a disaster is an event that brings injuries, disease, or contamination to people or may be linked to structural and physical damages without affecting public health and the health care system [1]. MCI is well-defined as an incident that overwhelms the local healthcare system, where the level of casualties notably exceeds the ability to face the healthcare needs of victims with local resources and capabilities in a short period [2–4]. More specifically, MCIs focus on the outstrips of the capacity of the first responders at the incident scene or the medical workers at the hospital to deliver optimal care to all victims at a time [1]. While almost all MCIs may be disasters that affect communities, not all disasters are considered as MCIs [1]. Hence, the incidents generating MCIs or disasters may be remarkably heterogeneous in nature, creating a

For peer review only - http://bmjopen.bmj.com/site/about/guidelines.xhtml

different level of severity and impact on the population, environment, rescue system, medical responders, and patient care management [5].

Large-scale incidences, including mass accidents and emergencies, result in mortality, morbidity, and severe physical, social, economic, and organizational damages [6]. Moreover, mass incidences have a significant detrimental effect on the physical and mental well-being of the exposed population [7]. For example, posttraumatic stress disorder (PTSD), depression, anxiety, and substance use are the most common mental conditions following a mass event or emergency [8, 9]. However, people exposed to mass incidents are not only victims of mental health conditions but also include family members, co-workers, and, most importantly, first responders and frontline healthcare workers [8, 10, 11].

Recent studies showed that rescuers and first responders, such as Emergency Medical Service (EMS) workers in prehospital and hospital settings, including firefighters, police and military personnel, emergency health workers, and mortuary staff reported adverse mental health conditions following MCIs and disasters [8]. Healthcare personnel, particularly emergency department (ED) workers, always encounter severely critical and demanding situations following a mass event. Consequently, healthcare workers met different stressors along with their occupational risk factors (for example, long working hours, heavy workload, poor working conditions, and sleep disturbance) and showed a greater likelihood of reporting adverse psychological health [12]. Moreover, healthcare workers, particularly nurses and doctors in emergency department, are more susceptible to psychological stress as they experience repetitive traumatic situations due to the nature of their work [8, 13].

First responders frequently worked under dangerous conditions where they had a greater risk for infectious diseases, adverse environmental exposures, traumatic injuries, and particularly, negative mental health outcomes from caring for severely injured persons, experiencing dead

For peer review only - http://bmjopen.bmj.com/site/about/guidelines.xhtml

bodies, body parts, treating someone who has lost their loved ones and dealing with unsuccessful efforts for the victims following a mass causality event [14]. For example, traumatic events, such as severe burns/injuries, suicide victims, or a child with unintentional injuries, positively correlate with peritraumatic distress and PTSD symptoms of nurses who work in the ED [15]. Similarly, a study in the Netherlands showed that nearly one out of three nurses in ED reported sub-clinical levels of depression, anxiety, and somatic symptoms, with more than eight percent meeting a clinical level of PTSD [16]. Moreover, another study on German physicians in ED reported that almost 8% of doctors had depressive symptoms, about 17% had probable PTSD, and more than 3% had clinical depressive symptoms [17]. Among Belgian emergency doctors, almost 15% had a clinical level of PTSD, about 11% had anxiety, and almost 8% had depression after traumatic incidents [18].

During MCIs, the ED became the epicenter of healthcare deliveries. Emergency nurses and doctors had a higher risk of reported mental outcomes than other healthcare workers, which calls for an investigation to explore the prevalence of mental health outcomes and their associated risk factors among nurses and doctors. Consequently, early detection of these adverse mental health conditions and risk factors would help in undertaking required actions that have important implications for the stress and mental health management of nurses and doctors. Moreover, some critical gaps in the existing literature must be addressed. Firstly, most previous reviews investigated either the psychological outcomes of all types of healthcare workers in general or used disasters and MCIs interchangeably [8, 19, 20], which demands special attention (i) for a particular group like nurses and doctors who play a critical role in response to any mass casualties, and (ii) for a distinctive investigation to measure the effect of MCIs on mental health. Secondly, much of the current research studied only PTSD as a significant mental health outcome following disasters and MCIs [21], which may overlook other potential mental health conditions, such as anxiety and depression, of healthcare workers.

For peer review only - http://bmjopen.bmj.com/site/about/guidelines.xhtml

Therefore, our study will answer the following research question: what are the levels of anxiety, depression, and PTSD and risk factors reported among emergency nurses and doctors in healthcare facilities and prehospital settings following mass causality incidents?

1.1 **Aims of the study**

Our study aims to summarize the current evidence on the levels of anxiety, depression, and PTSD and their associated risk factors among nurses and doctors in the hospital and prehospital setting following a mass causality incident.

2. Methods and analysis

2.1 Study design

This systematic review protocol is prepared following the Preferred Reporting Items for Systematic Reviews and Meta-Analysis Protocols (PRISMA-P) guidelines [22, 23] (Supplementary Table S1). To maintain complete transparency and reporting, particularly in the methods section used for identifying, selecting, and summarizing the eligible studies, we will follow the PRISMA-P statement and Narrative Synthesis Method [24]. The protocol has been registered at the International Prospective Register of Systematic Reviews (PROSPERO) with the registration number (CRD42023412852).

2.2 Eligibility criteria

The eligible studies will be peer-reviewed full-text English-language articles published between 1 January 2010 to 31 December 2022 that explored the prevalence and risk factors for any of the three mental health outcomes (anxiety, depression, or PTSD) among nurses and doctors in the hospital and prehospital setting during MCIs. Additionally, this review will include studies with quantitative, qualitative, or mixed methods research designs, and there

For peer review only - http://bmjopen.bmj.com/site/about/guidelines.xhtml

will be no restrictions on the geographical setting of the included studies. However, this review will exclude studies if they (i) include nurses and doctors who worked in the general department, (ii) include disasters or wars as exposure, (iii) include nurses and doctors who already have chronic mental disorders, or (iv) measure mental health outcomes without DSM-defined criteria, or (v) are other review articles, protocols, editorials, letter to editors, conference abstracts, commentaries, and opinion pieces (Table 1).

2.3 Information sources

The following seven databases will be systematically searched from 1 January 2010 to 31 December 2022 to retrieve relevant articles: PubMed, PsycINFO, MEDLINE Ovid, Embase, CINAHL, Web of Science, and Nursing & Allied Health database. These databases were also used to search the literature by the previous systematic reviews on healthcare workers' mass casualty or mental health outcomes. Moreover, this review will screen the references of all eligible articles to find possible further relevant studies due to literature saturation.

2.4 Search strategy

A systematic and detailed search strategy has been developed on PubMed/MEDLINE database using MESH terms and keywords. The search will use the Boolean operators (AND, OR, and NOT) and truncations (*) following the specifications of the database. In addition, the search strategy will be modified to fit other electronic databases depending on database-specific filters (Supplementary Table S2). The systematic search strategy includes keywords/terms related to the study's population, intervention, and outcomes of interest (Table 2). Moreover, the search terms will be finalized after consultation with experts in this field and a research librarian.

2.5 Study records

For peer review only - http://bmjopen.bmj.com/site/about/guidelines.xhtml

2.5.1 Screening procedures of eligible studies

After the initial systematic search, all the search records will be managed with Rayyans Qatar Computing Research Institute (QCRI) software, a web-based online tool for managing and reporting systematic reviews and meta-analyses [25]. All the retrieved articles will be stored in a single library to detect and remove duplicates. Then, a three-phase screening process will be followed to select eligible articles from the remaining articles. The first screening step will be assessing the relevancy of the article title to the review topic independently by two reviewers (H.U. and M.K.H.). Irrelevant studies will be excluded, and those that meet the review criteria will be kept. In the second step of screening, a pair of independent researchers (H.U. and M.K.H.) of the research team will conduct the abstract screening for eligibility of the remaining studies and exclude studies that do not meet the inclusion criteria. In the final step, the full text of the remained articles will be obtained and further assessed for inclusion by two reviewers (H.U. and M.K.H.). A third reviewer (R.C.D.) of the research team will be consulted to resolve any disagreement in screening titles, abstracts, and full-text articles. A flow diagram will be prepared to report the study selection process and reasons for exclusion following the PRISMA guidelines [26] (Figure 1).

2.5.2 Data extraction and management

À standardized data extraction template in an Excel sheet will be prepared, and two independent reviewers (H.U. and M.K.H.) will collect specific information from the eligible studies following an instruction manual. The data extraction and information items will be included based on the PICO structure [27]. For example, participant characteristics (gender, age, marital status, education, years of experience, type of work), intervention/exposure characteristics (types of mass casualty, frequency, duration, and the severity of the incident), and outcome characteristics (types, prevalence, and duration of the conditions) will be

For peer review only - http://bmjopen.bmj.com/site/about/guidelines.xhtml

extracted. Moreover, general information (e.g., article title, author, reviewer, record number, date of data extraction), study characteristics (aim/objective of the study, study design, countries, sample size), and the main findings from the eligible studies related to our research questions will be extracted. In case of missing information in eligible studies, an email will be sent to the corresponding authors for missing details. After the data extraction by two reviewers, a third reviewer will verify and resolve any disagreement if it happens.

2.5.3 Quality appraisal/Risk of bias

The methodological quality and risk of bias will be assessed for the finally included studies to examine whether each study addresses the required dimensions of research quality. The STROBE (Strengthening the Reporting of Observational Studies in Epidemiology) Checklist [28] for observational studies, the COREQ (Consolidated Criteria for Reporting Qualitative Research) Checklist for qualitative studies [29], and the Mixed Methods Appraisal tool (MMAT) [30] for mixed method studies will be used to measure the quality appraisal of the eligible studies. The overall quality of the studies will be rated as *poor*, *fair*, and *good* quality following the GRADE (Grading of Recommendations Assessment, Development, and Evaluation) approach [31]. Two reviewers (H.U. and M.K.H.) will independently evaluate the quality assessment of the included studies. Any discrepancies in the process and the grading of quality appraisal will be resolved through group discussion; if necessary, a third reviewer will settle any unresolved conflicts. After the quality evaluation, only the studies with *fair* and *good* categories will be considered for this review.

2.5.4 Data synthesis and analysis

After the full-text screening of the included studies, this review will explore the prevalence of the outcomes and their associated risk factors among the study population. As the eligible

For peer review only

studies are expected to be heterogeneous in terms of measurements of variables, methods, and results, this review will follow the narrative synthesis method along with using the key characteristics and findings of the studies. One of the benefits of narrative synthesis is to offer the opportunity to compare the findings of the eligible studies, particularly focusing on the population, intervention, and outcome. Based on the different healthcare settings (hospital vs prehospital), or different risk factors, subgroup analysis will be executed depending on data availability. Even if possible, this review will conduct a meta-analysis of the quantitative data. Any modification to the protocol considered by the research team will be well reported in the review with proper explanation.

2.6 Assessment of publication bias

Publication bias assessment will be performed statistically by conducting Egger's test and visually by producing funnel plots. This review does not consider examining the meta-bias or confidence in cumulative evidence.

2.7 Ethical considerations

This systematic review deals with existing published studies without any personally identifiable information of participants. Therefore, ethical approval from the research committee is not required.

2.8 Patient and public involvement

Patients or the public were not engaged in this research design, reporting, or dissemination plan.

3. Discussion

For peer review only - http://bmjopen.bmj.com/site/about/guidelines.xhtml

For peer review only

Many studies reported the adverse effect of mass emergencies on medical responders' mental health and well-being, where nurses and doctors reported a higher risk of being exposed to the outcomes [8]. Moreover, the prevalence and risk factors may vary based on the nature of the mass casualties. However, previous studies have documented the prevalence of mental health outcomes, particularly PTSD, among healthcare workers in different disasters. There is no systematic review on anxiety, depression, and PTSD among emergency nurses and doctors following MCIs. Therefore, this systematic review will fill the gap in the literature on the prevalence and the associated risk factors of anxiety, depression, and PTSD among nurses and doctors. The findings will offer vital information for public health stakeholders, healthcare managers, policymakers, and researchers to design and develop mental health management programs for emergency nurses and doctors.

6. Conclusions

The findings of this systematic review will offer a synthesis of current evidence on the prevalence and the associated risk factors of anxiety, depression, and PTSD among nurses and doctors following MCIs. These findings will help health organizations, managers, and all types of hospitals, regardless of geographical location, to adopt preventive measures and decrease adverse psychological outcomes among nurses and doctors after mass casualties.

Ethics and dissemination: This systematic review deals with existing published studies without any personally identifiable information of participants. Therefore, ethical approval from the research committee is not required. Findings from this review will be disseminated in peer-reviewed journals and presented at relevant international conferences.

For peer review only - http://bmjopen.bmj.com/site/about/guidelines.xhtml

Authors' contributions: H.U., M.K.H., and R.C.D. developed the idea and design for this protocol. H.U. wrote the first draft of the manuscript and developed the search strategy and methodology. R.C.D., and M.K.H. critically revised this manuscript. H.U. and M.K.H. will perform the study selection and data extraction. R.C.D. will supervise this study. All the authors critically revised the methodology and approved the final version of this manuscript.

Funding: This research received no external funding.

Competing interests: The authors declare that they have no competing interests.

List of abbreviations

MCI: Mass casualty incident, PTSD: Posttraumatic stress disorder, EMS: Emergency medical service, ED: Emergency department, PRISMA-P: Preferred reporting items for systematic reviews and meta-analysis protocols, PROSPERO: International prospective register of systematic reviews, QCRI: Rayyans qatar computing research institute, STROBE: Strengthening the reporting of observational studies in epidemiology, COREQ: Consolidated criteria for reporting qualitative research, MMAT: Mixed methods appraisal tool, GRADE: Grading of recommendations assessment, development, and evaluation

Authors' information: [1]Department of Global Public Health, Karolinska Institutet, Solna, 17177, Sweden; [2]Department of Medicine, University of Oviedo, 33006 Oviedo, Spain; [3]Department of Sociology, East West University, Dhaka 1212, Bangladesh; [4]Institute of Disaster Management and Vulnerability Studies, University of Dhaka, Dhaka 1000, Bangladesh; [5]Health Service of the Principality of Asturias (SAMU-Asturias), Health Research Institute of the Principality of Asturias (Research Group on Prehospital Care and Disasters, GIAPREDE), 33001 Oviedo, Spain.

For peer review only - http://bmjopen.bmj.com/site/about/guidelines.xhtml

References

1. Lynn M, Lieberman H, Lynn L, Pust GD, Stahl K, Yeh DD, et al., editors. Disasters and Mass Casualty Incidents: The Nuts and Bolts of Preparedness and Response to Protracted and Sudden Onset Emergencies. Cham: Springer International Publishing; 2019.

2. Doyle CJ. Mass casualty incident. Integration with prehospital care. Emerg Med Clin North Am. 1990;8:163–75.

3. Schenk E, Wijetunge G, Mann NC, Lerner EB, Longthorne A, Dawson D. Epidemiology of Mass Casualty Incidents in the United States. Prehosp Emerg Care. 2014;18:408–16.

4. WHO. Mass casualty management systems: Strategies and guidelines for building health sector capacity. Geneva (Switzerland): World Health Organization; 2007.

5. Gamberini L, Imbriaco G, Ingrassia PL, Mazzoli CA, Badiali S, Colombo D, et al. Logistic Red Flags in Mass-Casualty Incidents and Disasters: A Problem-Based Approach. Prehospital Disaster Med. 2022;37:197–204.

6. Makwana N. Disaster and its impact on mental health: A narrative review. J Fam Med Prim Care. 2019;8:3090.

7. Norris FH, Friedman MJ, Watson PJ, Byrne CM, Diaz E, Kaniasty K. 60,000 Disaster Victims Speak: Part I. An Empirical Review of the Empirical Literature, 1981—2001. Psychiatry. 2002;65:207–39.

8. Naushad VA, Bierens JJ, Nishan KP, Firjeeth CP, Mohammad OH, Maliyakkal AM, et al. A Systematic Review of the Impact of Disaster on the Mental Health of Medical Responders. Prehospital Disaster Med. 2019;34:632–43.

9. North CS, Nixon SJ, Shariat S, Mallonee S, Curtis McMillen J, Spitznagel EL, et al. Psychiatric disorders among survivors of the Oklahoma City bombing. In: The Science of Mental Health. Taylor and Francis; 2013. p. 159–67.

10. Palmer J, Ku M, Wang H, Crosse K, Bennett A, Lee E, et al. Public health emergency and psychological distress among healthcare workers: a scoping review. BMC Public Health. 2022;22:1396.

11. Spoorthy MS, Pratapa SK, Mahant S. Mental health problems faced by healthcare workers due to the COVID-19 pandemic–A review. Asian J Psychiatry. 2020;51:102119.

12. Lopes Cardozo B, Gotway Crawford C, Eriksson C, Zhu J, Sabin M, Ager A, et al. Psychological distress, depression, anxiety, and burnout among international humanitarian aid workers: a longitudinal study. PloS One. 2012;7:e44948.

13. Gillespie GL, Bresler S, Gates DM, Succop P. Posttraumatic Stress Symptomatology among Emergency Department Workers following Workplace Aggression. Workplace Health Saf. 2013;61:247–54.

For peer review only

14. van der Velden PG, van Loon P, Benight CC, Eckhardt T. Mental health problems among search and rescue workers deployed in the Haïti earthquake 2010: a pre-post comparison. Psychiatry Res. 2012;198:100–5.

15. Lavoie S, Talbot LR, Mathieu L, Dallaire C, Dubois M-F, Courcy F. An exploration of factors associated with posttraumatic stress in ER nurses. J Nurs Manag. 2016;24:174–83.

16. Adriaenssens J, de Gucht V, Maes S. The impact of traumatic events on emergency room nurses: findings from a questionnaire survey. Int J Nurs Stud. 2012;49:1411–22.

17. Pajonk F-GB, Cransac P, Müller V, Teichmann A, Meyer W. Trauma and stress-related disorders in German emergency physicians: the predictive role of personality factors. Int J Emerg Ment Health. 2012;14:257–68.

18. Somville FJ, De Gucht V, Maes S. The impact of occupational hazards and traumatic events among Belgian emergency physicians. Scand J Trauma Resusc Emerg Med. 2016;24:59.

19. Beaglehole B, Mulder RT, Frampton CM, Boden JM, Newton-Howes G, Bell CJ. Psychological distress and psychiatric disorder after natural disasters: systematic review and meta-analysis. Br J Psychiatry J Ment Sci. 2018;213:716–22.

20. Rodriguez-Arrastia M, García-Martín M, Villegas-Aguilar E, Ropero-Padilla C, Martin-Ibañez L, Roman P. Emotional and psychological implications for healthcare professionals in disasters or mass casualties: A systematic review. J Nurs Manag. 2022;30:298–309.

21. Neria Y, Nandi A, Galea S. Posttraumatic stress disorder following disasters: a systematic review. Psychol Med. 2008;38:467–80.

22. Moher D, Shamseer L, Clarke M, Ghersi D, Liberati A, Petticrew M, et al. Preferred reporting items for systematic review and meta-analysis protocols (PRISMA-P) 2015 statement. Syst Rev. 2015;4:1.

23. Shamseer L, Moher D, Clarke M, Ghersi D, Liberati A, Petticrew M, et al. Preferred reporting items for systematic review and meta-analysis protocols (PRISMA-P) 2015: elaboration and explanation. BMJ. 2015;350:g7647.

24. Popay J, Roberts H, Sowden A, Petticrew M, Arai L, Rodgers M, et al. Guidance on the conduct of narrative synthesis in systematic reviews. Prod ESRC Methods Programme Version. 2006;1:b92.

25. Ouzzani M, Hammady H, Fedorowicz Z, Elmagarmid A. Rayyan—a web and mobile app for systematic reviews. Syst Rev. 2016;5:210.

26. Moher D, Liberati A, Tetzlaff J, Altman DG, Group TP. Preferred Reporting Items for Systematic Reviews and Meta-Analyses: The PRISMA Statement. PLOS Med. 2009;6:e1000097.

27. Methley AM, Campbell S, Chew-Graham C, McNally R, Cheraghi-Sohi S. PICO, PICOS and SPIDER: a comparison study of specificity and sensitivity in three search tools for qualitative systematic reviews. BMC Health Serv Res. 2014;14:579.

For peer review only - http://bmjopen.bmj.com/site/about/guidelines.xhtml

28. Von Elm E, Altman DG, Egger M, Pocock SJ, Gøtzsche PC, Vandenbroucke JP. The Strengthening the Reporting of Observational Studies in Epidemiology (STROBE) statement: guidelines for reporting observational studies. The Lancet. 2007;370:1453–7.

29. Tong A, Sainsbury P, Craig J. Consolidated criteria for reporting qualitative research (COREQ): a 32-item checklist for interviews and focus groups. Int J Qual Health Care. 2007;19:349–57.

30. Pluye P, Gagnon M-P, Griffiths F, Johnson-Lafleur J. A scoring system for appraising mixed methods research, and concomitantly appraising qualitative, quantitative and mixed methods primary studies in Mixed Studies Reviews. Int J Nurs Stud. 2009;46:529–46.

31. Guyatt GH, Oxman AD, Vist GE, Kunz R, Falck-Ytter Y, Alonso-Coello P, et al. GRADE: an emerging consensus on rating quality of evidence and strength of recommendations. BMJ. 2008;336:924–6.

For peer review only

For peer review only - http://bmjopen.bmj.com/site/about/guidelines.xhtml

Table 1: Inclusion and exclusion criteria of the systematic review

	Inclusion criteria	Exclusion criteria
Population	Nurses and doctors worked in the emergency department of healthcare facilities and prehospital settings	Nurses and doctors in the general departments
Intervention	Mass casualty incidents (MCIs)	Disasters and wars
Comparison	Nurses vs doctors	
Outcome	Investigated the prevalence and risk factors for anxiety, depression, or PTSD (defined following DSM-III, DSM-III-R, DSM-IV, or DSM-V criteria)	Already chronic mental disorders, any other mental conditions, did not measure the DSM-defined symptoms
Study	All quantitative, qualitative, and mixed methods peer-reviewed studies, including RCTs, non-RCTs, cohort studies, cross-sectional studies, and surveys. The study must consider at least one outcome following MCIs	Protocols, editorials, letters to editors, commentaries, conference abstracts and posters, and opinion pieces that are not peer-reviewed, including grey literature
Setting	Irrespective of location and geography	
Date Range	1 January 2010 to 31 December 2022	Published before January 2010 and after December 2022
Language	English	Studies published in languages other than English

For peer review only - http://bmjopen.bmj.com/site/about/guidelines.xhtml

Table 2: key terms for preparing the comprehensive search strategy

	Key terms
# Population	nurse, emergency nurse, prehospital nurse, registered nurse, intern nurse, nurse in emergency department, physician, doctor, medical doctor, emergency doctor, prehospital doctor, medical officer, doctor in the emergency department
# Intervention/ exposure	mass casualty incident, MCI, explosion, plane crash, air crash, air accident, train derailment, train bombing, road traffic, bus bombing, bus crash, car crash, suicide bombing, OR bombing, terror attack, terrorism, bioterrorism, accident, industrial accident, fire, factory fire, chemical spill, CBRNE incident, building collapse, mass injury, gunshot, mass shooting, massive chemical contamination, radiological dispersal device (RDD), dirty bomb, emergency, outbreak, COVID-19, pandemic, volcanic eruptions, earthquake
# Outcome	anxiety, depression, posttraumatic stress disorder, PTSD, mental health disorder, psychological disorder, psychological condition, psychological distress, psychological impact, mental outcome, mental condition, emotional impacts

For peer review only

Figure 1: Study selection flow diagram (PRISMA 2009)

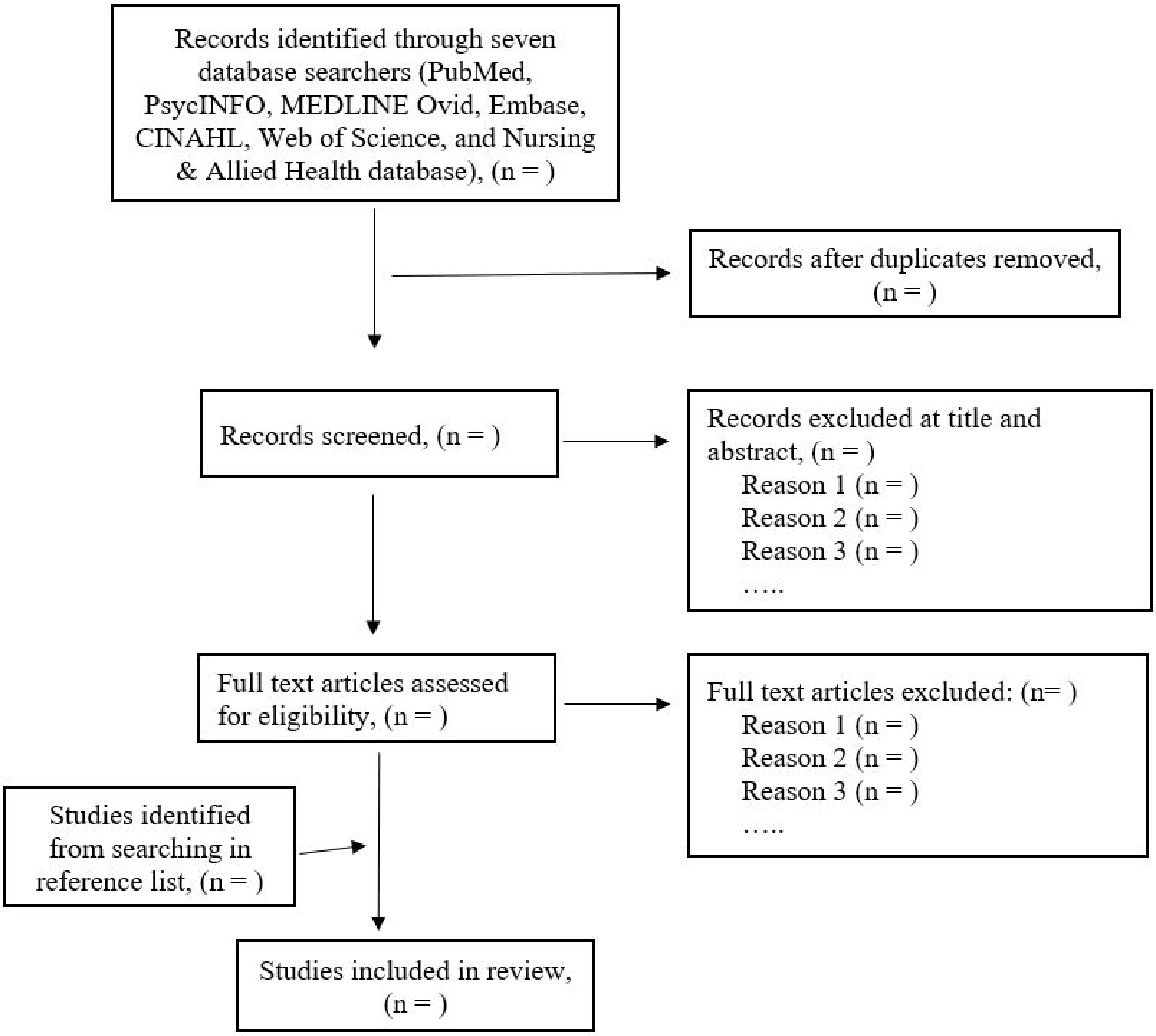

only

For peer review only - http://bmjopen.bmj.com/site/about/guidelines.xhtml

Supplementary Table

Table S1: PRISMA-P Statement – Checklist of items

Section and topic	Item No	Page number of manuscript
**ADMINISTRATIVE INFORMATION**		
Title:		
Identification	1a	1
Update	1b	N/A
Registration	2	2
Authors:		
Contact	3a	1
Contributions	3b	12
Amendments	4	N/A
Support:		
Sources	5a	6-7
Sponsor	5b	12
Role of sponsor or funder	5c	12
**INTRODUCTION**		
Rationale	6	3-6
Objectives	7	6
**METHODS**		
Eligibility criteria	8	6-7
Information sources	9	7
Search strategy	10	7
Study records:		
Data management	11a	8
Selection process	11b	8-9
Data collection process	11c	8-9
Data items	12	9-10
Outcomes and prioritization	13	6-7
Risk of bias in individual studies	14	9
Data synthesis	15a-15d	9-10
Meta-bias(es)	16	10
Confidence in cumulative evidence	17	10

For peer review only - http://bmjopen.bmj.com/site/about/guidelines.xhtml

Table S2: Comprehensive search strategy in PubMed

Sl no.	Search queries
#1	"Mass Casualty Incidents"[Mesh]) OR (MCI[Title/Abstract])) OR (explosion*[Title/Abstract])) OR (plane crash*[Title/Abstract])) OR (air crash*[Title/Abstract])) OR (air accident*[Title/Abstract])) OR (train derailment[Title/Abstract])) OR (train bombing[Title/Abstract])) OR (road traffic[Title/Abstract])) OR (bus bombing[Title/Abstract])) OR (bus crash*[Title/Abstract])) OR (car crash*[Title/Abstract])) OR (suicide bombing[Title/Abstract])) OR (bombing[Title/Abstract])) OR (terror attack[Title/Abstract])) OR (terrorism[Title/Abstract])) OR (bioterrorism[Title/Abstract])) OR (accident*[Title/Abstract])) OR (industrial accident*[Title/Abstract])) OR (fire*[Title/Abstract])) OR (factory fire*[Title/Abstract])) OR (chemical spill*[Title/Abstract])) OR (CBRNE accident*[Title/Abstract])) OR (building collapse[Title/Abstract])) OR (mass injur*[Title/Abstract])) OR (gunshot*[Title/Abstract])) OR (mass shoot*[Title/Abstract])) OR (massive chemical contamination[Title/Abstract])) OR (radiological dispersal device[Title/Abstract])) OR (dirty bomb*[Title/Abstract])) OR ("Disease Outbreaks"[Mesh])) OR (emergenc*[Title/Abstract])) OR ("COVID-19"[Mesh])) OR (volcanic eruption*[Title/Abstract]) OR ("Earthquakes"[Mesh])
# 2	"Stress Disorders, Post-Traumatic"[Mesh]) OR (PTSD[Title/Abstract])) OR ("Anxiety"[Mesh])) OR ("Anxiety Disorders"[Mesh])) OR ("Patient Health Questionnaire"[Mesh])) OR ("Depression"[Mesh])) OR ("Depressive Disorder"[Mesh])) OR ("Mental Health"[Mesh])) OR (mental health disorder*[Title/Abstract])) OR (psychological disorder*[Title/Abstract])) OR (psychological condition*[Title/Abstract])) OR (psychological distres*[Title/Abstract])) OR (psychological impact*[Title/Abstract])) OR (mental health outcome*[Title/Abstract])) OR (mental condition*[Title/Abstract])) OR (emotional impact*[Title/Abstract])
#3	"Nurses"[Mesh]) OR ("Nurses, Community Health"[Mesh])) OR ("Nurses, Public Health"[Mesh])) OR (emergency nurse*[Title/Abstract])) OR (prehospital nurse*[Title/Abstract])) OR (registered nurse*[Title/Abstract])) OR (intern nurse*[Title/Abstract])) OR (nurse in emergency department[Title/Abstract])) OR ("Physicians"[Mesh])) OR (medical doctor*[Title/Abstract])) OR (doctor*[Title/Abstract])) OR (emergency doctor*[Title/Abstract])) OR (prehospital doctor*[Title/Abstract])) OR (medical officer*[Title/Abstract])) OR (doctor in emergency department[Title/Abstract])
# 4	#1 AND #2 AND #3 AND
# 5	#1 AND #2 AND #3 AND Filter: English, Full text, Humans, from 2010 – 2022

BMJ Open

Effects of Mass Casualty Incidents on Anxiety, Depression, and PTSD among Doctors and Nurses: A Systematic Review Protocol

Journal:	*BMJ Open*
Manuscript ID	bmjopen-2023-075478.R1
Article Type:	Protocol
Date Submitted by the Author:	10-Aug-2023
Complete List of Authors:	Uddin, Helal; Karolinska Institute, Global Public Health ; East West University,<br>Hasan, Md. Khalid; Dhaka University<br>castro delgado, rafael; University of Oviedo, Medicine. Public Health; SAMU-Asturias, Emergency Medicine
**Primary Subject Heading**:	Mental health
Secondary Subject Heading:	Nursing, Public health
Keywords:	Anxiety disorders < PSYCHIATRY, Depression & mood disorders < PSYCHIATRY, Physicians, MENTAL HEALTH

SCHOLARONE™
Manuscripts

Effects of Mass Casualty Incidents on Anxiety, Depression, and PTSD among Doctors and Nurses: A Systematic Review Protocol

Helal Uddin[1,2,3], Md. Khalid Hasan[4], and Rafael Castro Delgado [1,5]

[1]Department of Global Public Health, Karolinska Institutet, Solna, 17177, Sweden

[2]Department of Medicine, University of Oviedo, 33006 Oviedo, Spain

[3]Department of Sociology, East West University, Dhaka 1212, Bangladesh

[4]Institute of Disaster Management and Vulnerability Studies, University of Dhaka, Dhaka 1000, Bangladesh

[5]Health Service of the Principality of Asturias (SAMU-Asturias), Health Research Institute of the Principality of Asturias (Research Group on Prehospital Care and Disasters, GIAPREDE), 33001, Oviedo, Spain

Author Note:

Helal Uddin https://orcid.org/0000-0002-0767-3174

Md. Khalid Hasan https://orcid.org/0000-0002-9293-9693

Rafael Castro Delgado https://orcid.org/0000-0001-9520-656X

Corresponding Author:

Helal Uddin, Department of Global Public Health, Karolinska Institutet, Solna, 17177, Sweden. Email: md.helal.uddin@stud.ki.se; helal@ewubd.edu

&

Rafael Castro Delgado, Health Service of the Principality of Asturias (SAMU-Asturias), Health Research Institute of the Principality of Asturias (Research Group on Prehospital Care and Disasters, GIAPREDE), 33001, Oviedo, Spain. Email: castrorafael@uniovi.es; rafacastrosamu@yahoo.es

Word count: Text=2433, Abstract =246

Number of tables & figures: Table 2, Figure 1, Supplementary Table 2

For peer review only - http://bmjopen.bmj.com/site/about/guidelines.xhtml

For peer review only

Abstract

Introduction: Both doctors and nurses showed a greater risk of being exposed to different mental health conditions following mass casualties. This systematic review aims to synthesize the existing evidence on the prevalence of anxiety, depression, and PTSD and their associated risk factors among doctors and nurses following mass casualty incidents.

Methods and analysis: Seven electronic databases (PubMed, PsycINFO, MEDLINE Ovid, Embase, CINAHL, Web of Science, and Nursing & Allied Health database) will be searched from 2010 to 2022 with peer-reviewed articles in English language using the pre-defined keywords. Two reviewers will independently screen the titles and abstracts, as well as review the full texts using the eligibility criteria, then extract data independently. The National Institutes of Health Quality Assessment Tools (NIH-QAT) for quantitative studies, the Critical Appraisal Skills Programme (CASP) Checklist for qualitative studies, and the Mixed Methods Appraisal tool (MMAT) for mixed method studies will be used to measure the quality appraisal of eligible studies. A third reviewer will resolve the discrepancies when the two reviewers cannot reach an agreement in any step. The result from the eligible studies will be described following narrative synthesis with the key characteristics and findings of the included studies, and meta-analysis will be performed if applicable.

Ethics and dissemination: This systematic review deals with existing published studies without any personally identifiable information of participants. Therefore, ethical approval from the research committee is not required. Findings from this review will be disseminated in peer-reviewed journals and presented at relevant international conferences.

PROSPERO registration number: CRD42023412852

Keywords: mass casualty incident, anxiety, depression, PTSD, nurse, doctor

For peer review only - http://bmjopen.bmj.com/site/about/guidelines.xhtml

Strengths and limitations

- To the best knowledge, this is the first systematic review on two crucial medical responders (emergency doctors and nurses), and their three mental health outcomes (anxiety, depression, and PTSD) following MCIs.
- Another biggest strength of this review is that it follows the PRISMA guidelines strictly and uses standard quality assessment tools.
- Above and beyond, the review will incorporate studies irrespective of location or geography.
- However, excluding gray literature and including studies published only in English will miss some potential studies that may be a source of publication bias.
- As different authors use heterogeneous tools to measure the outcomes, there will be a possibility of reporting bias.

1. Introduction

Mass casualty incidents (MCIs), and disasters are frequently used interchangeably in the literature, although they describe specific conditions and entities. From a medical perspective, a disaster is an event that brings injuries, disease, or contamination to people or may be linked to structural and physical damages without affecting public health and the health care system [1]. MCI is well-defined as an incident that overwhelms the local healthcare system, where the level of casualties notably exceeds the ability to face the healthcare needs of victims with local resources and capabilities in a short period [2–4]. More specifically, MCIs focus on the outstrips of the capacity of the first responders at the incident scene or the medical workers at the hospital to deliver optimal care to all victims at a time [1]. While almost all MCIs may be disasters that affect communities, not all disasters are considered as MCIs [1]. Hence, the incidents generating MCIs or disasters may be remarkably heterogeneous in nature, creating a

For peer review only - http://bmjopen.bmj.com/site/about/guidelines.xhtml

For peer review only

different level of severity and impact on the population, environment, rescue system, medical responders, and patient care management [5].

Large-scale incidents, including mass accidents and emergencies, result in mortality, morbidity, and severe physical, social, economic, and organizational damages [6]. Moreover, mass incidents have a significant detrimental effect on the physical and mental well-being of the exposed population [7]. For example, posttraumatic stress disorder (PTSD), depression, anxiety, and substance use are the most common mental conditions following a mass event or emergency [8, 9]. However, people exposed to mass incidents are not only victims of mental health conditions but also include family members, co-workers, and, most importantly, first responders and frontline healthcare workers [8, 10, 11].

Recent studies showed that rescuers and first responders, such as Emergency Medical Service (EMS) workers in prehospital and hospital settings, including firefighters, police and military personnel, emergency health workers, and mortuary staff reported adverse mental health conditions following MCIs and disasters [8]. Healthcare personnel, particularly emergency department (ED) workers, always encounter severely critical and demanding situations following a mass event. Consequently, healthcare workers met different stressors along with their occupational risk factors (for example, long working hours, heavy workload, poor working conditions, and sleep disturbance) and showed a greater likelihood of reporting adverse psychological health [12]. Moreover, healthcare workers, particularly nurses, and doctors in the emergency department, are more susceptible to psychological stress as they experience repetitive traumatic situations due to the nature of their work [8, 13].

First responders frequently worked under dangerous conditions where they had a greater risk for infectious diseases, adverse environmental exposures, traumatic injuries, and particularly, negative mental health outcomes from caring for severely injured persons, experiencing dead

For peer review only - http://bmjopen.bmj.com/site/about/guidelines.xhtml

For peer review only

bodies, body parts, treating someone who has lost their loved ones and dealing with unsuccessful efforts for the victims following a mass causality event [14]. For example, traumatic events, such as severe burns/injuries, suicide victims, or a child with unintentional injuries, positively correlate with peritraumatic distress and PTSD symptoms of nurses who work in the ED [15]. Similarly, a study in the Netherlands showed that nearly one out of three nurses in ED reported sub-clinical levels of depression, anxiety, and somatic symptoms, with more than eight percent meeting a clinical level of PTSD [16]. Moreover, another study on German physicians in ED reported that almost 8% of doctors had depressive symptoms, about 17% had probable PTSD, and more than 3% had clinical depressive symptoms [17]. Among Belgian emergency doctors, almost 15% had a clinical level of PTSD, about 11% had anxiety, and almost 8% had depression after traumatic incidents [18].

During MCIs, the ED became the epicenter of healthcare deliveries. Emergency doctors and nurses had a higher risk of reported mental outcomes than other healthcare workers, which calls for an investigation to explore the prevalence of mental health outcomes and their associated risk factors among doctors and nurses. Consequently, early detection of these adverse mental health conditions and risk factors would help in undertaking required actions that have important implications for the stress and mental health management of nurses and doctors. Moreover, some critical gaps in the existing literature must be addressed. Firstly, most previous reviews investigated either the psychological outcomes of all types of healthcare workers in general or used disasters and MCIs interchangeably [8, 19, 20], which demands special attention (i) for a particular group like doctors and nurses who play a critical role in response to any mass casualties, and (ii) for a distinctive investigation to measure the effect of MCIs on mental health. Secondly, much of the current research studied only PTSD as a significant mental health outcome following disasters and MCIs [21], which may overlook other potential mental health conditions, such as anxiety and depression, of healthcare workers. Therefore, our

For peer review only - http://bmjopen.bmj.com/site/about/guidelines.xhtml

study will answer the following research question: What are the levels of anxiety, depression, and PTSD and their associated risk factors reported among emergency doctors and nurses in healthcare facilities and prehospital settings following mass causality incidents?

1.1 **Aims of the study**

Our study aims to summarize the current evidence on the prevalence of anxiety, depression, and PTSD and their associated risk factors among doctors and nurses in the hospital and prehospital setting following a mass causality incident.

2. Methods and analysis

2.1 Study design

This systematic review protocol is prepared following the Preferred Reporting Items for Systematic Reviews and Meta-Analysis Protocols (PRISMA-P) guidelines [22, 23] (Supplementary Table S1). To maintain complete transparency and reporting, particularly in the methods section used for identifying, selecting, and summarizing the eligible studies, we will follow the PRISMA-P statement and Narrative Synthesis Method [24]. The protocol has been registered at the International Prospective Register of Systematic Reviews (PROSPERO) with the registration number (CRD42023412852).

2.2 Eligibility criteria

The eligible studies will be peer-reviewed full-text English-language articles published between 1 January 2010 to 31 December 2022 that explored the prevalence and risk factors for any of the three mental health outcomes (anxiety, depression, or PTSD) among nurses and doctors in the hospital and prehospital setting during MCIs. Additionally, this review will include studies with quantitative, qualitative, or mixed methods research designs, and there

For peer review only - http://bmjopen.bmj.com/site/about/guidelines.xhtml

will be no restrictions on the geographical setting of the included studies. However, this review will exclude studies if they (i) include nurses and doctors who worked in the general department, (ii) include disasters or wars as exposure, (iii) include nurses and doctors who already have chronic mental disorders, or (iv) measure mental health outcomes without DSM and ICD-10, and ICD-11-defined criteria, or (v) are other review articles, protocols, editorials, letter to editors, conference abstracts, commentaries, and opinion pieces (Table 1).

2.3 Information sources

The following seven databases will be systematically searched from 1 January 2010 to 31 December 2022 to retrieve relevant articles: PubMed, PsycINFO, MEDLINE Ovid, Embase, CINAHL, Web of Science, and Nursing & Allied Health database. These databases were also used to search the literature by the previous systematic reviews on healthcare workers' mass casualty or mental health outcomes. Moreover, this review will screen the references of all eligible articles to find possible further relevant studies due to literature saturation.

2.4 Search strategy

A systematic and detailed search strategy has been developed on PubMed/MEDLINE database using MESH terms and keywords. The search will use the Boolean operators (AND, OR, and NOT) and truncations (*) following the specifications of the database. In addition, the search strategy will be modified to fit other electronic databases depending on database-specific filters (Supplementary Table S2). The systematic search strategy includes keywords/terms related to the study's population, intervention, and outcomes of interest (Table 2). Moreover, the search terms will be finalized after consultation with experts in this field and a research librarian.

2.5 Study records

For peer review only - http://bmjopen.bmj.com/site/about/guidelines.xhtml

2.5.1 Screening procedures of eligible studies

After the initial systematic search, all the search records will be managed with Rayyans Qatar Computing Research Institute (QCRI) software, a web-based online tool for managing and reporting systematic reviews and meta-analyses [25]. All the retrieved articles will be stored in a single library to detect and remove duplicates. Then, a three-phase screening process will be followed to select eligible articles from the remaining articles. The first screening step will be assessing the relevancy of the article title to the review topic independently by two reviewers (H.U. and M.K.H.). Irrelevant studies will be excluded, and those that meet the review criteria will be kept. In the second step of screening, a pair of independent researchers (H.U. and M.K.H.) of the research team will conduct the abstract screening for eligibility of the remaining studies and exclude studies that do not meet the inclusion criteria. In the final step, the full text of the remained articles will be obtained and further assessed for inclusion by two reviewers (H.U. and M.K.H.). A third reviewer (R.C.D.) of the research team will be consulted to resolve any disagreement in screening titles, abstracts, and full-text articles. A flow diagram will be prepared to report the study selection process and reasons for exclusion following the PRISMA guidelines [26] (Figure 1).

2.5.2 Data extraction and management

À standardized data extraction template in an Excel sheet will be prepared, and two independent reviewers (H.U. and M.K.H.) will collect specific information from the eligible studies following an instruction manual. The data extraction and information items will be included based on the PICO structure [27]. For example, participant characteristics (gender, age, marital status, education, years of experience, type of work), intervention/exposure characteristics (types of mass casualty, frequency, duration, and the severity of the incident), and outcome characteristics (types, prevalence, and duration of the conditions) will be

For peer review only - http://bmjopen.bmj.com/site/about/guidelines.xhtml

extracted. Moreover, general information (e.g., article title, author, reviewer, record number, date of data extraction), study characteristics (aim/objective of the study, study design, countries, sample size), and the main findings from the eligible studies related to our research questions will be extracted. In case of missing information in eligible studies, an email will be sent to the corresponding authors for missing details. After the data extraction by two reviewers, a third reviewer will verify and resolve any disagreement if it happens.

2.5.3 Quality appraisal/Risk of bias

The methodological quality and risk of bias will be assessed for the finally included studies to examine whether each study addresses the required dimensions of research quality. The National Institutes of Health Quality Assessment Tools (NIH-QAT) for quantitative studies [28] and the Critical Appraisal Skills Programme (CASP) checklist for qualitative studies [29], and the Mixed Methods Appraisal tool (MMAT) [30] for mixed method studies will be used to measure the quality appraisal of the eligible studies. The overall quality of the studies will be rated as *poor*, *fair*, and *good* quality following the GRADE (Grading of Recommendations Assessment, Development, and Evaluation) approach [31]. Two reviewers (H.U. and M.K.H.) will independently evaluate the quality assessment of the included studies. Any discrepancies in the process and the grading of quality appraisal will be resolved through group discussion; if necessary, a third reviewer will settle any unresolved conflicts. After the quality evaluation, only the studies with *fair* and *good* categories will be considered for this review.

2.5.4 Data synthesis and analysis

After the full-text screening of the included studies, this review will explore the prevalence of the outcomes and their associated risk factors among the study population. As the eligible

For peer review only - http://bmjopen.bmj.com/site/about/guidelines.xhtml

studies are expected to be heterogeneous in terms of measurements of variables, methods, and results, this review will follow the narrative synthesis method along with using the key characteristics and findings of the studies. One of the benefits of narrative synthesis is to offer the opportunity to compare the findings of the eligible studies, particularly focusing on the population, intervention, and outcome. Based on the different healthcare settings (hospital vs. prehospital), or different risk factors, subgroup analysis will be executed depending on data availability. Even if possible, this review will conduct a meta-analysis of the quantitative data. Any modification to the protocol considered by the research team will be well reported in the review with proper explanation.

2.6 Assessment of publication bias

Publication bias assessment will be performed statistically by conducting Egger's test and visually by producing funnel plots. This review does not consider examining the meta-bias or confidence in cumulative evidence.

2.7 Ethical considerations

This systematic review deals with existing published studies without any personally identifiable information of participants. Therefore, ethical approval from the research committee is not required.

2.8 Patient and public involvement

Patients or the public were not engaged in this research design, reporting, or dissemination plan.

3. Discussion

For peer review only - http://bmjopen.bmj.com/site/about/guidelines.xhtml

Many studies reported the adverse effect of mass emergencies on medical responders' mental health and well-being, where nurses and doctors reported a higher risk of being exposed to the outcomes [8]. Moreover, the prevalence and risk factors may vary based on the nature of the mass casualties. However, previous studies have documented the prevalence of mental health outcomes, particularly PTSD, among healthcare workers in different disasters. There is no systematic review on anxiety, depression, and PTSD among emergency doctors and nurses following MCIs. Therefore, this systematic review will fill the gap in the literature on the prevalence and the associated risk factors of anxiety, depression, and PTSD among nurses and doctors. The findings will offer vital information for public health stakeholders, healthcare managers, policymakers, and researchers to design and develop mental health management programs for emergency doctors and nurses.

6. Conclusions

The findings of this systematic review will offer a synthesis of current evidence on the prevalence and the associated risk factors of anxiety, depression, and PTSD among doctors and nurses following MCIs. These findings will help health organizations, managers, and all types of hospitals, regardless of geographical location, to adopt preventive measures and decrease adverse psychological outcomes among nurses and doctors after mass casualties.

Ethics and dissemination:

This systematic review deals with existing published studies without any personally identifiable information of participants. Therefore, ethical approval from the research committee is not required. Findings from this review will be disseminated in peer-reviewed journals and presented at relevant international conferences.

For peer review only - http://bmjopen.bmj.com/site/about/guidelines.xhtml

Authors' contributions:

H.U., M.K.H., and R.C.D. developed the idea and design for this protocol. H.U. wrote the first draft of the manuscript and developed the search strategy and methodology. R.C.D., and M.K.H. critically revised this manuscript. H.U. and M.K.H. will perform the study selection and data extraction. R.C.D. will supervise this study. All the authors critically revised the methodology and approved the final version of this manuscript.

Funding:

This research received no external funding.

Competing interests:

The authors declare that they have no competing interests.

List of abbreviations:

MCI: Mass casualty incident,

PTSD: Posttraumatic stress disorder,

EMS: Emergency medical service,

ED: Emergency department,

PRISMA-P: Preferred reporting items for systematic reviews and meta-analysis protocols,

PROSPERO: International prospective register of systematic reviews,

QCRI: Rayyans qatar computing research institute,

NIH-QAT: National Institutes of Health Quality Assessment Tools,

CASP: Critical Appraisal Skills Programme,

MMAT: Mixed methods appraisal tool,

For peer review only

GRADE: Grading of recommendations assessment, development, and evaluation

Authors' information:

[1]Department of Global Public Health, Karolinska Institutet, Solna, 17177, Sweden; [2]Department of Medicine, University of Oviedo, 33006 Oviedo, Spain; [3]Department of Sociology, East West University, Dhaka 1212, Bangladesh; [4]Institute of Disaster Management and Vulnerability Studies, University of Dhaka, Dhaka 1000, Bangladesh; [5]Health Service of the Principality of Asturias (SAMU-Asturias), Health Research Institute of the Principality of Asturias (Research Group on Prehospital Care and Disasters, GIAPREDE), 33001 Oviedo, Spain.

For peer review only

References

1. Lynn M, Lieberman H, Lynn L, Pust GD, Stahl K, Yeh DD, et al., editors. Disasters and Mass Casualty Incidents: The Nuts and Bolts of Preparedness and Response to Protracted and Sudden Onset Emergencies. Cham: Springer International Publishing; 2019.

2. Doyle CJ. Mass casualty incident. Integration with prehospital care. Emerg Med Clin North Am. 1990;8:163–75.

3. Schenk E, Wijetunge G, Mann NC, Lerner EB, Longthorne A, Dawson D. Epidemiology of Mass Casualty Incidents in the United States. Prehosp Emerg Care. 2014;18:408–16.

4. WHO. Mass casualty management systems: Strategies and guidelines for building health sector capacity. Geneva (Switzerland): World Health Organization; 2007.

5. Gamberini L, Imbriaco G, Ingrassia PL, Mazzoli CA, Badiali S, Colombo D, et al. Logistic Red Flags in Mass-Casualty Incidents and Disasters: A Problem-Based Approach. Prehospital Disaster Med. 2022;37:197–204.

6. Makwana N. Disaster and its impact on mental health: A narrative review. J Fam Med Prim Care. 2019;8:3090.

7. Norris FH, Friedman MJ, Watson PJ, Byrne CM, Diaz E, Kaniasty K. 60,000 Disaster Victims Speak: Part I. An Empirical Review of the Empirical Literature, 1981—2001. Psychiatry. 2002;65:207–39.

8. Naushad VA, Bierens JJ, Nishan KP, Firjeeth CP, Mohammad OH, Maliyakkal AM, et al. A Systematic Review of the Impact of Disaster on the Mental Health of Medical Responders. Prehospital Disaster Med. 2019;34:632–43.

9. North CS, Nixon SJ, Shariat S, Mallonee S, Curtis McMillen J, Spitznagel EL, et al. Psychiatric disorders among survivors of the Oklahoma City bombing. In: The Science of Mental Health. Taylor and Francis; 2013. p. 159–67.

10. Palmer J, Ku M, Wang H, Crosse K, Bennett A, Lee E, et al. Public health emergency and psychological distress among healthcare workers: a scoping review. BMC Public Health. 2022;22:1396.

11. Spoorthy MS, Pratapa SK, Mahant S. Mental health problems faced by healthcare workers due to the COVID-19 pandemic–A review. Asian J Psychiatry. 2020;51:102119.

12. Lopes Cardozo B, Gotway Crawford C, Eriksson C, Zhu J, Sabin M, Ager A, et al. Psychological distress, depression, anxiety, and burnout among international humanitarian aid workers: a longitudinal study. PloS One. 2012;7:e44948.

13. Gillespie GL, Bresler S, Gates DM, Succop P. Posttraumatic Stress Symptomatology among Emergency Department Workers following Workplace Aggression. Workplace Health Saf. 2013;61:247–54.

For peer review only - http://bmjopen.bmj.com/site/about/guidelines.xhtml

14. van der Velden PG, van Loon P, Benight CC, Eckhardt T. Mental health problems among search and rescue workers deployed in the Haïti earthquake 2010: a pre-post comparison. Psychiatry Res. 2012;198:100–5.

15. Lavoie S, Talbot LR, Mathieu L, Dallaire C, Dubois M-F, Courcy F. An exploration of factors associated with post-traumatic stress in ER nurses. J Nurs Manag. 2016;24:174–83.

16. Adriaenssens J, de Gucht V, Maes S. The impact of traumatic events on emergency room nurses: findings from a questionnaire survey. Int J Nurs Stud. 2012;49:1411–22.

17. Pajonk F-GB, Cransac P, Müller V, Teichmann A, Meyer W. Trauma and stress-related disorders in German emergency physicians: the predictive role of personality factors. Int J Emerg Ment Health. 2012;14:257–68.

18. Somville FJ, De Gucht V, Maes S. The impact of occupational hazards and traumatic events among Belgian emergency physicians. Scand J Trauma Resusc Emerg Med. 2016;24:59.

19. Beaglehole B, Mulder RT, Frampton CM, Boden JM, Newton-Howes G, Bell CJ. Psychological distress and psychiatric disorder after natural disasters: systematic review and meta-analysis. Br J Psychiatry J Ment Sci. 2018;213:716–22.

20. Rodriguez-Arrastia M, García-Martín M, Villegas-Aguilar E, Ropero-Padilla C, Martin-Ibañez L, Roman P. Emotional and psychological implications for healthcare professionals in disasters or mass casualties: A systematic review. J Nurs Manag. 2022;30:298–309.

21. Neria Y, Nandi A, Galea S. Post-traumatic stress disorder following disasters: a systematic review. Psychol Med. 2008;38:467–80.

22. Moher D, Shamseer L, Clarke M, Ghersi D, Liberati A, Petticrew M, et al. Preferred reporting items for systematic review and meta-analysis protocols (PRISMA-P) 2015 statement. Syst Rev. 2015;4:1.

23. Shamseer L, Moher D, Clarke M, Ghersi D, Liberati A, Petticrew M, et al. Preferred reporting items for systematic review and meta-analysis protocols (PRISMA-P) 2015: elaboration and explanation. BMJ. 2015;350:g7647.

24. Popay J, Roberts H, Sowden A, Petticrew M, Arai L, Rodgers M, et al. Guidance on the conduct of narrative synthesis in systematic reviews. Prod ESRC Methods Programme Version. 2006;1:b92.

25. Ouzzani M, Hammady H, Fedorowicz Z, Elmagarmid A. Rayyan—a web and mobile app for systematic reviews. Syst Rev. 2016;5:210.

26. Moher D, Liberati A, Tetzlaff J, Altman DG, Group TP. Preferred Reporting Items for Systematic Reviews and Meta-Analyses: The PRISMA Statement. PLOS Med. 2009;6:e1000097.

27. Methley AM, Campbell S, Chew-Graham C, McNally R, Cheraghi-Sohi S. PICO, PICOS and SPIDER: a comparison study of specificity and sensitivity in three search tools for qualitative systematic reviews. BMC Health Serv Res. 2014;14:579.

For peer review only - http://bmjopen.bmj.com/site/about/guidelines.xhtml

28. National Heart, Lung, and Blood Institute. Study Quality Assessment Tools | NHLBI, NIH. 2021. https://www.nhlbi.nih.gov/health-topics/study-quality-assessment-tools. Accessed 9 Jul 2023.

29. CASP. CASP Checklists - Critical Appraisal Skills Programme. CASP - Critical Appraisal Skills Programme. 2018. https://casp-uk.net/casp-tools-checklists/. Accessed 15 Jul 2023.

30. Pluye P, Gagnon M-P, Griffiths F, Johnson-Lafleur J. A scoring system for appraising mixed methods research, and concomitantly appraising qualitative, quantitative and mixed methods primary studies in Mixed Studies Reviews. Int J Nurs Stud. 2009;46:529–46.

31. Guyatt GH, Oxman AD, Vist GE, Kunz R, Falck-Ytter Y, Alonso-Coello P, et al. GRADE: an emerging consensus on rating quality of evidence and strength of recommendations. BMJ. 2008;336:924–6.

For peer review only

For peer review only - http://bmjopen.bmj.com/site/about/guidelines.xhtml

Table 1: Inclusion and exclusion criteria of the systematic review

	**Inclusion criteria**	**Exclusion criteria**
Population	Nurses and doctors worked in the emergency department of healthcare facilities and prehospital settings	Nurses and doctors in the general departments
Intervention	Mass casualty incidents (MCIs)	Disasters and wars
Comparison	Nurses vs doctors	
Outcome	Investigated the prevalence and risk factors for anxiety, depression, or PTSD (defined following DSM-III, DSM-III-R, DSM-IV, or DSM-V and ICD-10 and ICD-11 criteria)	Already chronic mental disorders, any other mental conditions, did not measure the DSM and ICD-defined symptoms
Study	All quantitative, qualitative, and mixed methods peer-reviewed studies, including RCTs, non-RCTs, cohort studies, cross-sectional studies, and surveys. The study must consider at least one outcome following MCIs	Protocols, editorials, letters to editors, commentaries, conference abstracts and posters, and opinion pieces that are not peer-reviewed, including grey literature
Setting	Irrespective of location and geography	
Date Range	1 January 2010 to 31 December 2022	Published before January 2010 and after December 2022
Language	English	Studies published in languages other than English

For peer review only - http://bmjopen.bmj.com/site/about/guidelines.xhtml

Table 2: key terms for preparing the comprehensive search strategy

	Key terms
# Population	nurse, emergency nurse, prehospital nurse, registered nurse, intern nurse, nurse in emergency department, physician, doctor, medical doctor, emergency doctor, prehospital doctor, medical officer, doctor in the emergency department
# Intervention/ exposure	mass casualty incident, MCI, explosion, plane crash, air crash, air accident, train derailment, train bombing, road traffic, bus bombing, bus crash, car crash, suicide bombing, OR bombing, terror attack, terrorism, bioterrorism, accident, industrial accident, fire, factory fire, chemical spill, CBRNE incident, building collapse, mass injury, gunshot, mass shooting, massive chemical contamination, radiological dispersal device (RDD), dirty bomb, emergency, volcanic eruptions, earthquake
# Outcome	anxiety, depression, posttraumatic stress disorder, PTSD, mental health disorder, psychological disorder, psychological condition, psychological distress, psychological impact, mental outcome, mental condition, emotional impacts

For peer review only - http://bmjopen.bmj.com/site/about/guidelines.xhtml

Figure 1: Study selection flow diagram (PRISMA 2009)

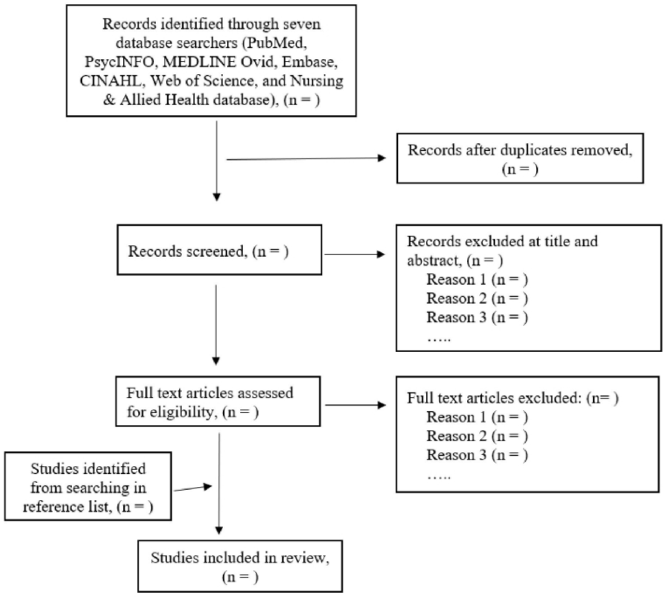

Figure 1

215x279mm (300 x 300 DPI)

Supplementary Table

Table S1: PRISMA-P Statement – Checklist of items

Section and topic	Item No	Page number of manuscript
**ADMINISTRATIVE INFORMATION**		
Title:		
Identification	1a	1
Update	1b	N/A
Registration	2	2
Authors:		
Contact	3a	1
Contributions	3b	12
Amendments	4	N/A
Support:		
Sources	5a	6-7
Sponsor	5b	12
Role of sponsor or funder	5c	12
**INTRODUCTION**		
Rationale	6	3-6
Objectives	7	6
**METHODS**		
Eligibility criteria	8	6-7
Information sources	9	7
Search strategy	10	7
Study records:		
Data management	11a	8
Selection process	11b	8-9
Data collection process	11c	8-9
Data items	12	9-10
Outcomes and prioritization	13	6-7
Risk of bias in individual studies	14	9
Data synthesis	15a-15d	9-10
Meta-bias(es)	16	10
Confidence in cumulative evidence	17	10

For peer review only

Table S2: Comprehensive search strategy in PubMed

Sl no.	Search queries
#1	"Mass Casualty Incidents"[Mesh]) OR (MCI[Title/Abstract])) OR (explosion*[Title/Abstract])) OR (plane crash*[Title/Abstract])) OR (air crash*[Title/Abstract])) OR (air accident*[Title/Abstract])) OR (train derailment[Title/Abstract])) OR (train bombing[Title/Abstract])) OR (road traffic[Title/Abstract])) OR (bus bombing[Title/Abstract])) OR (bus crash*[Title/Abstract])) OR (car crash*[Title/Abstract])) OR (suicide bombing[Title/Abstract])) OR (bombing[Title/Abstract])) OR (terror attack[Title/Abstract])) OR (terrorism[Title/Abstract])) OR (bioterrorism[Title/Abstract])) OR (accident*[Title/Abstract])) OR (industrial accident*[Title/Abstract])) OR (fire*[Title/Abstract])) OR (factory fire*[Title/Abstract])) OR (chemical spill*[Title/Abstract])) OR (CBRNE accident*[Title/Abstract])) OR (building collapse[Title/Abstract])) OR (mass injur*[Title/Abstract])) OR (gunshot*[Title/Abstract])) OR (mass shoot*[Title/Abstract])) OR (massive chemical contamination[Title/Abstract])) OR (radiological dispersal device[Title/Abstract])) OR (dirty bomb*[Title/Abstract])) OR (emergenc*[Title/Abstract])) OR (volcanic eruption*[Title/Abstract]) OR ("Earthquakes"[Mesh])
# 2	"Stress Disorders, Post-Traumatic"[Mesh]) OR (PTSD[Title/Abstract])) OR ("Anxiety"[Mesh])) OR ("Anxiety Disorders"[Mesh])) OR ("Patient Health Questionnaire"[Mesh])) OR ("Depression"[Mesh])) OR ("Depressive Disorder"[Mesh])) OR ("Mental Health"[Mesh])) OR (mental health disorder*[Title/Abstract])) OR (psychological disorder*[Title/Abstract])) OR (psychological condition*[Title/Abstract])) OR (psychological distres*[Title/Abstract])) OR (psychological impact*[Title/Abstract])) OR (mental health outcome*[Title/Abstract])) OR (mental condition*[Title/Abstract])) OR (emotional impact*[Title/Abstract])
#3	"Nurses"[Mesh]) OR ("Nurses, Community Health"[Mesh])) OR ("Nurses, Public Health"[Mesh])) OR (emergency nurse*[Title/Abstract])) OR (prehospital nurse*[Title/Abstract])) OR (registered nurse*[Title/Abstract])) OR (intern nurse*[Title/Abstract])) OR (nurse in emergency department[Title/Abstract])) OR ("Physicians"[Mesh])) OR (medical doctor*[Title/Abstract])) OR (doctor*[Title/Abstract])) OR (emergency doctor*[Title/Abstract])) OR (prehospital doctor*[Title/Abstract])) OR (medical officer*[Title/Abstract])) OR (doctor in emergency department[Title/Abstract])
# 4	#1 AND #2 AND #3 AND
# 5	#1 AND #2 AND #3 AND Filter: English, Full text, Humans, from 2010 – 2022
